# Early Parity Epigenetic Footprint of *FOXA1* Gene Body in Normal Breast Tissue of Iranian Women

**DOI:** 10.29252/.23.2.99

**Published:** 2019-03

**Authors:** Zahra Zendehbad, Pantea Izadi, Abdolreza Daraei, Mir Saeed Yekaninejad, Nahid Nafissi, Nasim Younosi, Ghasemali Khorasani, Javad Tavakkoly Bazzaz

**Affiliations:** 1 Department of Medical Genetics, School of Medicine, Tehran University of Medical Sciences, Tehran, Iran;; 2 Genetic Department, Faculty of Medicine, Babol University of Medical Sciences, Babol, Iran;; 3 Department of Epidemiology and Biostatistics, School of Public Health, Tehran University of Medical Sciences, Tehran, Iran;; 4 Surgical Department, School of Medicine, Iran University of Medical Science, Tehran, Iran;; 5 Surgical Department, School of Medicine, Shahid Beheshti University of Medical Sciences, Tehran, Iran;; 6 Division of Plastic and Reconstructive Surgery, Imam Khomeini Hospital Complex, Tehran University of Medical Sciences, Tehran, Iran

**Keywords:** Breast cancer, DNA methylation, Epigenetics, FOXA1, Pregnancy

## Abstract

**Background:**

Young age at first full-term pregnancy (FFTP) is an important factor in breast cancer risk reduction. It is postulated that this protective effect is the result of stable molecular signatures imprinted by physiological process of pregnancy, but the molecular mechanism of this protective role is unclear. The aim of the current study was to identify the effect of early FFTP on methylation status of *FOXA1* gene body. FOXA1 is an essential transcription factor for mammary gland development and estrogen responsiveness of breast tissue.

**Methods:**

Fresh frozen normal breast tissues (n = 51) were collected from Iranian women who underwent cosmetic mammoplasty (27 nulliparous women and 24 parous women who have experienced first pregnancy before the age of 25). DNA was extracted and then methylated DNA immunoprecipitation (MeDIP) real-time PCR was used to assess *FOXA1* gene body methylation.

**Results:**

Our results revealed that *FOXA1* methylation level is significantly higher in early parous compared with nulliparous group (*p *= 0.041).

**Conclusion:**

Our study provides new hint about the association between early FFTP and epigenetic modifications within gene body of *FOXA1* in normal breast tissue. More investigation is required for clarifying molecular mechanisms underlying this association in order to develop breast cancer prevention strategies.

## INTRODUCTION

Breast cancer is the most common cancer in women worldwide. This malignancy has various risk factors that are divided into modifiable and non-modifiable factors. An early first full-term pregnancy (FFTP) is the most effective modifiable and natural prevention method against breast cancer that can decrease women’s lifelong risk up to 50%^ [^^1^^]^.

The first documented scrutiny of this preventive performance was conducted by Bernardino Ramazzini, the father of industrial medicine, in 18^th^ century. He noted that “the nuns are found with breast tumors more than any other women”, and he deduced this because nuns remain celibate and childless in their lifetime^[^^2^^]^.

Major evidence that displayed a correlation between parity and decreased breast cancer risk was through MacMahon *et al.*'s^[^^1^^]^ research, in which they carried out an international collaborative case-control study. They found that pregnancy at early age associates with protection against breast cancer. Women who experienced their first pregnancy before 20 years of age had a 50% reduced risk of breast cancer compared with nulliparous women^[^^1^^]^. Postponing first delivery after 35 years of age had a 23% increased breast cancer risk^[^^3^^]^. The early pregnancy protective effect is predominantly against estrogen receptor (ER) positive breast tumors^[^^4^^]^ and this protection is negligible for first gestations, which occur between the age of 30 to 34 years^[^^1^^,^^5^^,^^6^^]^. A lot of epidemiological studies have shown that further gestations and breastfeeding increase the mentioned protective effect^[^^7^^-^^9^^]^. It is important to consider this protection because most women in different countries, especially in Western communities, have been shown to remain nullipara or to postpone the first pregnancy till the age of 35^[^^10^^,^^11^^]^. Therefore, the recognition of possible molecular mechanisms involved in protective effect of pregnancy will stimulate the advancement of new cancer prevention strategies. 

Several animal models studies have revealed that physiological process of early FFTP can induce particular molecular signatures in normal breast tissue. Researchers have demonstrated that these molecular changes play an important role in full differentiation of mammary glands and finally result in lifelong breast cancer prevention^[^^12^^-^^16^^]^. In addition to the complete differentiation of breast tissue, it has been suggested that FFTP protective effect may occur through three ways, including parity-specific changes in the levels of circulating hormones, reduction in the number of mammary stem cells, and variation in response to estrogen in normal breast tissue^[^^4^^]^. Despite extensive studies, the molecular mechanisms of parity-associated protective effect remain unclear. The lifelong breast cancer protection requires persistent molecular changes in the genome of mammary cells; hence, we hypothesize that epigenetic alterations, which are stable and permanent, may play a role in pregnancy-associated protective effect. A previous survey has revealed that the breast epithelial nuclei of postmenopausal parous women are small and heterochromatic with the high level of histone methylation, as compared to nulliparous females^[^^12^^]^. Therefore, since 2014, researchers have focused on the investigation of parity-associated epigenetic alterations in the breast tissue of human and mice. For instance, Ghosh *et al.*^[^^17^^]^ have studied the epigenetic modifications of the normal breast tissue and found some parity-specific hypermethylated and hypo-methylated genes. *FOXA1* was an important hypermethylated gene among their findings. 

FOXA1 is a pioneer transcription factor for mammary gland development and has a significant function in the biology of luminal epithelial cells of the breast tissue^[^^18^^,^^19^^]^, also it is critical for the complete function of ERα, which is essential for hormone responsiveness of mammary tissue^[^^18^^,^^20^^]^. It has been shown that FOXA1 co-occupies ERα enhancers in the genome and regulates the expression of ERα downstream target genes^[^^18^^,^^21^^]^. It has also been explained that the lack of *FOXA1* expression leads to decreased breast cancer cell proliferation^[^^22^^]^.

In a preliminary research, we revealed the significant association between ESR1 methylation and some reproductive factors such as FFTP in breast tumors^[^^23^^]^. Since molecular status and molecular interactions in tumor tissues can be modified by malignancy process, which finally result in differences between malignant and normal tissues, in the next step, we evaluated this association in normal breast tissue with a different quantitative methylation assay, methylated DNA immunoprecipitation (MeDIP). According to our results, there was no relation between FFTP and ESR1 methylation levels in normal breast tissue^[24]^. Therefore, in the current study, we focused on *FOXA1* as an upstream regulator of ER function, and the methylation status of *FOXA1* gene was investigated in normal breast tissue specimens of early parous and nulliparous women, in order to identify specifically the effect of early parity on gene body methylation of *FOXA1*.

## MATERIALS AND METHODS

**Samples collection and DNA extraction**

 In this cross-sectional study, 51 normal breast tissue samples were collected from women who were mammoplasty candidates without a positive history of breast cancer or other forms of malignant diseases. The present work was approved by the local Ethical Committee of Tehran University of Medical Sciences (Tehran, Iran), and all participants signed the informed consent form. They also filled out a questionnaire regarding age, BMI (kg/m^2^), reproductive factors (the age at FFTP, number of pregnancies, breastfeeding duration), as well as medical and smoking history. Fresh frozen normal breast tissues were collected from Mehr-e-Sina Surgical Center, Sohrevardi Surgery Center, and Vali-e-Asr Clinic in Imam Khomeini Hospital in Tehran. Samples were divided into two groups: parous (n = 24) and nulliparous women (n = 27) and were stored at -80 °C, until DNA extraction was accomplished. Genomic DNA was extracted using the high salt method^[25]^. Isolated DNA was used as a template for MeDIP-quantitative polymerase chain reaction (qPCR) process.

**Table1:** Primer sequences for real-time PCR

**Gene**	**Primers sequence**	**Product size (bp)**
*H19* (positive control)	F: 5^'^- CAGGTCGGGCATTATCCAC-3^'^ R: 5^'^- GCTGTCCTTAGACGGAGTCG-3^'^	175
*GAPDH* (negative control)	F: 5^'^- CCACATCGCTCAGACACCAT-3^'^ R: 5^'^- CCCCCATACGACTGCAAAGA-3^'^	144
*FOXA1*	F: 5^'^-GTCCATAGGTGATTTGCTCTATCA-3^'^ R: 5^'^- CAGGAAGATGTGTAATCGCCTTA-3^'^	168

**MeDIP real-time qPCR**

**Fig. 1:**
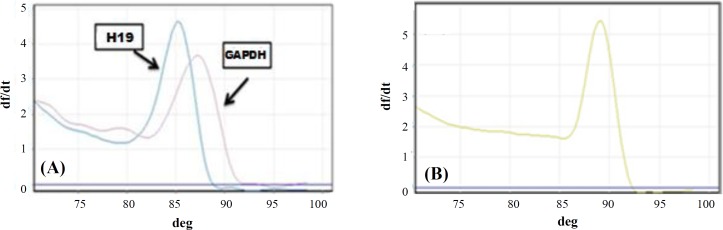
Melting curves analysis for (A) *GAPDH*, *H19* and (B) *FOXA1* after real-time PCR

**Fig. 2:**
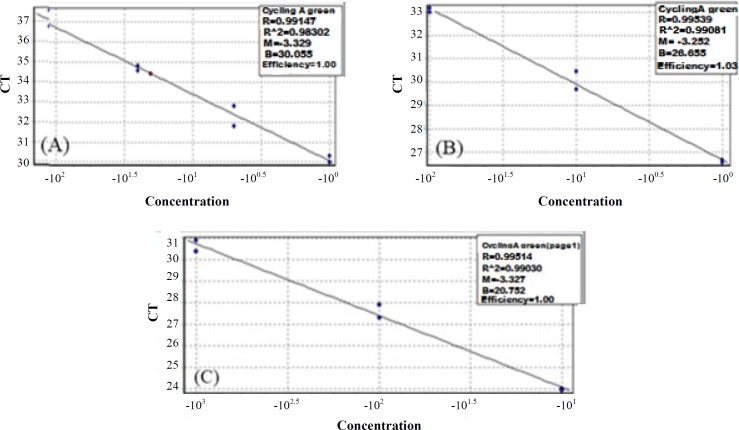
Standard curve for (A) *H19* (methylated control gene), (B) *GAPDH* (un-methylated control gene), and (C) *FOXA1* (target gene) real-time PCR assay for evaluating the reaction efficiency by serial dilutions of DNA (the reaction efficiency was acceptable)

 MeDIP was carried out using Methylamp^TM^ Methylated DNA capture kit )Epigentek, USA, Cat. No # P-1015). After extraction, DNA (1 μg) was sheared by sonication to obtain fragments ranging from 200 to 1000 bp. DNA was denatured at 95 °C for 5 minutes and was then divided into input (IN) and immunoprecipitated fractions. The immunoprecipitated fraction was incubated with anti-5^′^-Methylcytosine monoclonal antibody at room temperature for 1 hour and subsequently, was washed twice with 150 μl of antibody buffer and then with wash buffer. Finally, DNA was eluted from the column according to the kit instructions. Quality control of MeDIP and relative fold enrichment was performed by qPCR reaction using TAKARA SYBR Premix Ex Taq II (Tli Plus) (USA, Cat. No: RR820Q) by Corbett rotor gene 6000 cycler (Corbett Life Science, USA). To carry out these steps, two pairs of primers were designed to detect CpG islands of H19 imprinted control region (which is methylated) and GAPDH promoter (an unmethylated housekeeping gene) as MeDIP positive and negative controls, respectively (Table1). URPD online software (http://bio.kuas.edu.tw/urpd/) was employed to design the primers. The thermal cycling started with an initial step at 95 °C for 40 seconds to activate the hot-start DNA polymerase. Subsequently, a two-step cycling profile was done as follows: 40 cycles of annealing/ extension at 95 °C for 15 seconds and 60 °C for 35 seconds. To confirm the amplification of the PCR target region, the amplification step was followed by a melting cycle from 70 °C to 99 °C (Fig. 1). Serial dilution analysis was performed on different DNA concentrations to calculate the PCR efficiency, and the slope of the standard curve reactions (Fig. 2). Samples were tested in duplicate, and non-template control (NTC) samples (no template control; all reagents were used in qPCR except DNA) were also used in all reactions to rule out contamination. Finally, fold-enrichment ratio and specificity for each sample were calculated. When specificity was higher than 95% and fold-enrichment ratio exceeded 25^[^^26^^]^, MeDIP was considered successful.

**Fig. 3:**
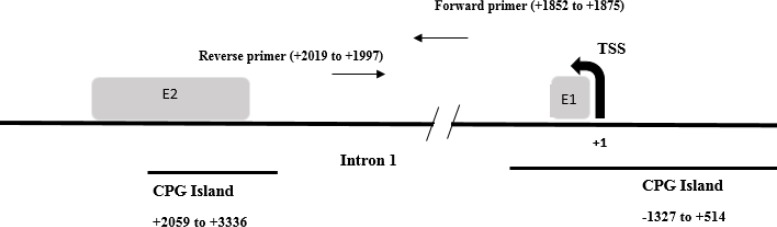
Graphical representation of *FOXA1* primers position. Primers are located in intron1 (reverse strand; +73 to +2262 relative to TSS). Amplified part contains eight gene body CpG sites (+1877, +1894, +1903, +1934, +1941, +1956, +1993, and +2002 relative to TSS)^[17]^. The two CPG islands sequences were extracted from University of California, Santa Cruz UCSC. TSS, transcription start site; E, exon

After accomplishing quality control for MeDIP, the enriched DNA fragments in MeDIP were quantified for target gene (*FOXA1*) by real-time qPCR (Tables 2). The primer sequences are shown in Table1, and Figure 3 shows the location of *FOXA1*-amplified region. Real-time PCR assay was performed in duplicate reactions and in the total volume of 20 μl. The thermal conditions of qPCR reaction were as follows: 95° C for 40 s, 95 °C for 15 s, 60 °C for 35 s (40 cycles). Relative enrichment of target locus was normalized by positive control (*H19* gene) and calculated with the 2^-∆∆ct^ formula.

**Statistical analysis**

 Data were represented by median and interquartile range or frequency and percentage. Non-parametric Mann-Whitney U test was used to compare *FOXA1* relative methylation between early parous and nulliparous groups. Kruskal-Wallis test was applied to investigate association between *FOXA1* methylation with breastfeeding duration and BMI. The association between *FOXA1* methylation and other factors (age, smoking, and number of pregnancies) were analyzed by Mann-Whitney U test. Moreover, non-parametric spearman correlation test was employed to calculate the correlation between *FOXA1* methylation and both age and breastfeeding durations. All analyzes were done by IBM SPSS Statistics version 22. Two tailed *p *values of less than 0.05 were considered statistically significant.

## RESULTS

**Fig 4:**
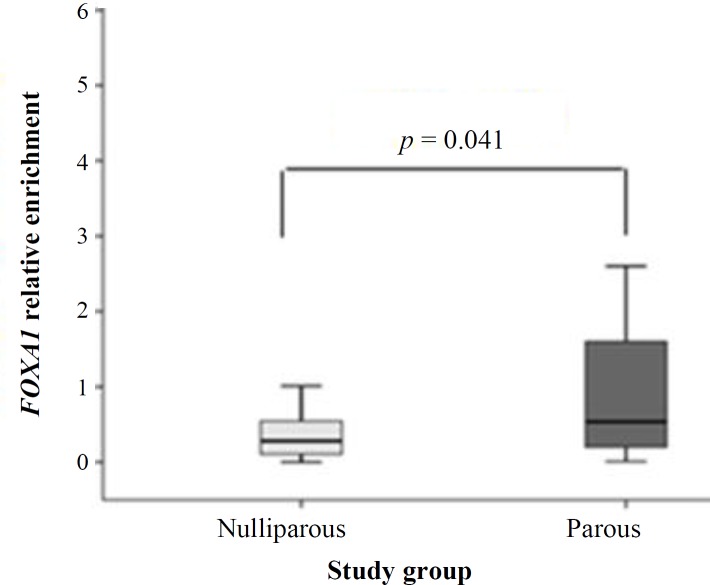
Comparison of *FOXA1* gene body methylation between parous and nulliparous groups. *FOXA1* methylation was significantly higher in parous group compared with nulliparous (*p* = 0.041)

 In this study, we used qPCR following MeDIP to compare *FOXA1* gene body methylation levels in normal breast tissue of early parous with nulliparous women. The MeDIP fold enrichment ratio and the specificity for all samples were in acceptable range; the means of them were 14663 and 98%, respectively, showing that MeDIP was performed successfully, and *H19*, as a methylated gene, was enriched 14663 times more than *GAPDH*, as an unmethylated control gene. The first group was early parous women (n = 24; mean age = 36.37 years) who had experienced their first full-term pregnancy under 25 years, and the second group was nulliparous women (n = 27; mean age = 31.92 years) who had never experienced pregnancy. All participants in this study were cancer-free Iranian women. The differences in methylation levels of studied subgroups are presented in Table 3. According to Whitney U test results, it was found that *FOXA1* relative methylation level in parous group is significantly higher than nulliparous (*p *= 0.041), as indicated in Figure 4. Median of *FOXA1* methylation in parous group was around twofold higher than nulliparous women (parous and nulliparous women median = 0.53 and 0.28, respectively). 

**Table 2:** Comparison of mean ∆ct values of control gene (*H19*) against unmethylated control gene (*GAPDH*) and target gene (*FOXA1*) against methylated control gene (*H19*)

Study groups	***H19*** ** mean ∆ct value** **(IP)** **(ct *H19***_IP_**–ct *GAPDHIP*)**	***H19*** ** mean ∆ct value** **(IN)** **(ct *H19***_IN_**–ct *GAPDH***_ IN_**)**	***FOXA1*** ** mean ∆ct value** **(IP)** **(ct *FOXA1***_IP_**–ct *H19***_IP_**)**	***FOXA1*** ** mean ∆ct value** **(IN)** **(ct *FOXA1***_IN_**–ct *H19***_IN_**)**
Parous (n = 24)	-9.63	0.88	4.15	2.98
Nulliparous (n = 27)	-7.94	0.66	6.18	3.96

In MeDIP technique for each reaction, DNA was sonicated into fragments ranging in size from 200-1000 bp and was divided into immunoprecipitated (IP) and input (IN) portions.

**Table 3:** Relative methylation levels among subgroups of study

**Variables**	**Number (%)**	**Relative methylation levels**		
		**Median**	**Q1-Q3**	***p *** **value**
Parity status				
Early parous Nulliparous	24 (47) 27 (53)	0.53 0.28	0.19-1.6 0.09-0.57	**0.041**
Age (year)				
<40 ≥40	41 (80) 10 (20)	0.38 0.34	0.13-0.91 0.14-0.75	0.79
BMI (kg/m^2^)				
<25 25-30 ≥30	24 (47.06) 20 (39.22) 7 (13.72)	0.46 0.33 0.08	0.16-0.84 0.17-0.71 0.01-0.95	0.44
Breastfeeding duration (month)				
Non-breastfed ≤12 12-24 >24	2 (8.33) 1 (4.17) 4 (16.67) 17 (70.83)	0.1 2.6 0.35 0.75	0.01 2.6-2.6 0.13-0.72 0.37-1.7	0.11
Number of full term pregnancies				
1 delivery 2 deliveries	11 (45.83) 13 (54.17)	0.54 0.51	0.21-2.06 0.18-1.19	0.56
Smoking				
Yes No	10 (19.6) 41 (80.4)	0.30 0.35	0.16-1.15 0.13-0.77	0.66

*p *value in bold represents a significant difference in the methylation level of the target regions between/among subgroups. Q: interquartile range

We found no association of reproductive (breastfeeding duration and number of deliveries) and non-reproductive factors (BMI, age, and smoking) with *FOXA1* gene body methylation; the results of these analyses are displayed in Table 3. Moreover, we did not observe any correlation between *FOXA1* methylation and both age and breastfeeding duration through spearman's correlation test.

## DISCUSSION

 Early age at FFTP is a known modifiable and a natural protective factor against breast cancer in women worldwide. Although this phenomenon was recognized many years ago^[^^1^^]^, the exact molecular mechanisms underlying this effect has remained unclear. Researchers hope to prevent or postpone the breast cancer in women at risk such as nulliparous women by some molecular interventions. This prevention policy requires discovering and understanding molecular changes that occur with early parity in normal breast tissue of women and lead to breast cancer protection.

 DNA methylation is a kind of epigenetic change that can be inducible and stable with a strong link to lifestyle-dependent features, such as BMI^[^^24^^]^ and parity^[^^17^^]^. Hence, it is possible that these lifelong epigenetic alterations, which are influenced by women’s lifestyle, can play a crucial role in the protective effect of pregnancy^[^^27^^]^. According to previous studies, the effect of early pregnancy on hormone responsiveness of breast tissue, especially estrogen hormone, is one of the proposed mechanisms of parity-associated breast cancer protection^[^^4^^]^. Therefore, we assume that studying the effects of early parity on some genes such as ESR1 and* FOXA1*, which have important functions in estrogen responsiveness^[^^18^^]^, can improve the better understanding of underlying mechanisms of early pregnancy associated with breast cancer protective effect. 

In our two previous studies, we showed that ESR1 methylation status has a significant association with FFTP in the breast tumors, but we did not find this association in normal breast tissues^[^^23^^,^^24^^]^. This result is consistent with the notion that the malignancy process can affect molecular status in tumor cells and make them different from normal cells. Following these investigations, in the present study, we decided to focus on the influence of early pregnancy on *FOXA1* gene methylation, which is an upstream regulator of ERS1 gene. Evidence from one study on normal breast tissue samples of a small group of American women (19 parous vs. 16 nulliparous) has shown the association of early FFTP with gene body hypermethylation of *FOXA1* by MBD-Cap technique^[^^17^^]^. In the current study, for investigating these new findings in a different larger study population, we evaluated 51 normal breast tissue samples of Iranian women with another quantitative epigenetic method (MeDIP-qPCR), which is more efficient for the investigation of gene body methylation in comparison with MBD-Cap method^[^^28^^]^. Our findings demonstrated that early parity has significant correlation with gene body hypermethylation of *FOXA1*. This finding is compatible with the results from the previous study^[^^17^^]^. 

FOXA1 is a pioneer transcription factor that co-localizes with ERα at enhancers in the genome and also is essential for ERα’s function^[^^18^^]^. This gene has high expression levels in luminal progenitor cells of the normal breast tissue, and their epigenetic status can be modified by early pregnancy^[^^19^^,^^29^^]^. Moreover, it has been shown that early parity is related to *FOXA1* epigenetic modification in epigenome mammary of mouse models^[^^30^^]^. In this case, researchers have shown that epigenetic modifications increase in *FOXA1* gene during mice gestation, which in turn correlates with down-regulation of *FOXA1* mRNA expression^[^^30^^]^. Thus, according to these findings^[^^30^^]^, which are in line with our results, it seems that *FOXA1* gene hypermethylarion is a new candidate epimark that is modified by early pregnancy in normal breast tissue. Regarding the protective role of early FFTP against ERα^+ ^breast tumors^[^^4^^,^^31^^]^ and its influence on methylation status of *FOXA1* gene (as an upstream regulator of ERα)^[^^18^^]^, it can be hypothesized that early pregnancy may change estrogen responsiveness of normal mammary tissue and result in protection against breast cancer. 

Epidemiological studies have shown that reproductive factors such as prolonged breastfeeding duration and increased number of births can lead to reduction in the risk of breast cancer^[^^7^^-^^9^^]^. Hence, we attempted to investigate whether these reproductive factors could alter *FOXA1* gene body methylation in normal breast tissue or not. Regarding our results, there was no association between these factors and *FOXA1* methylation levels. It seems that the protective effect of lactation and the increased number of births against breast cancer do not act through the same molecular mechanisms of early FFTP. 

Based on our results, non-reproductive factors such as BMI, age, and smoking do not have any significant association with *FOXA1* methylation. It seems that non-reproductive factors cannot influence the epigenetic status of *FOXA1* gene in normal breast tissue. However, regarding our limited sample size, these results should be interpreted with caution and are required to be clarified in future investigations with a larger sample size.

In conclusion, it seems that early parity, as an important breast cancer protective factor, can induce epigenetic changes in normal breast tissue. Also, all protective reproductive factors perhaps do not have similar effect on epigenetic status of normal breast tissue and may reduce the risk of breast cancer through different mechanisms. Our study provides a new hint about the association between early FFTP and epigenetic alterations within gene body of *FOXA1* in normal breast tissue. Because of limited sample size and lack of *FOXA1* expression analysis in this research, more investigation is needed to clarify the molecular mechanisms underlying this association with the aim of identifying promising new ways to advance breast cancer prevention strategies^[^^32^^]^.

## References

[1] MacMahon B, Cole P, Lin TM, Lowe CR, Mirra AP, Ravnihar B, Salber EJ, Valaoras VG, Yuasa S (1970). Age at first birth and breast cancer risk. Bulletin of the world health organization.

[2] Mustacchi P (1961). Ramazzini and Rigoni-Stern on parity and breast cancer: Clinical impression and statistical corroboration. Archives of internal medicine.

[3] Mathews T, Hamilton BE (2016). Mean Age of Mothers is on the Rise: United States, 2000-2014. NCHS data brief.

[4] Britt K, Ashworth A, Smalley M (2007). Pregnancy and the risk of breast cancer. Endocrine-related cancer.

[5] Barton M, Santucci-Pereira J, Russo J (2014). Molecular pathways involved in pregnancy-induced prevention against breast cancer. Frontiers in endocrinology.

[6] Meier-Abt F, Bentires-Alj M, Rochlitz C (2015). Breast cancer prevention: Lessons to be learned from mechanisms of early pregnancy-mediated breast cancer protection. Cancer research.

[7] Kotsopoulos J, Lubinski J, Salmena L, Lynch HT, Kim-Sing C, Foulkes WD, Ghadirian P, Neuhausen SL, Demsky R, Tung N, Ainsworth P, Senter L, Eisen A, Eng C, Singer C, Ginsburg O, Blum J, Huzarski T, Poll A, Sun P, Narod SA, Hereditary Breast Cancer Clinical Study Group (2012). Breastfeeding and the risk of breast cancer in BRCA1 and BRCA2 mutation carriers. Breast cancer research.

[8] De Silva M, Senarath U, Gunatilake M, Lokuhetty D (2010). Prolonged breastfeeding reduces risk of breast cancer in Sri Lankan women: A case-control study. Cancer epidemiology.

[9] Ma H, Henderson KD, Sullivan-Halley J, Duan L, Marshall SF, Ursin G, Horn-Ross PL, Largent J, Deapen DM, Lacey JV, Bernstein L (2010). Pregnancy-related factors and the risk of breast carcinoma in situ and invasive breast cancer among postmenopausal women in the California Teachers Study cohort. Breast cancer research.

[10] Livingston G, Cohn D U.S. birth rate falls to a record low; decline is greatest among immigrants.

[11] Australian Institute of Health and Welfare Breast Cancer in Australia: An Overview.

[12] Russo J, Santucci‐Pereira J, de Cicco RL, Sheriff F, Russo PA, Peri S, Slifker M, Ross E, Mello ML, Vidal BC, Belitskaya‐Lévy I, Arslan A, Zeleniuch-Jacquotte A, Bordas P, Lenner P, Ahman J, Afanasyeva Y, Hallmans G, Toniolo P, Russo IH (2012). Pregnancy‐induced chromatin remodeling in the breast of postmenopausal women. International journal of cancer.

[13] Russo J, Russo I (1997). Role of differentiation in the pathogenesis and prevention of breast cancer. Endocrine-related cancer.

[14] Russo I, Koszalka M, Russo J (1991). Comparative study of the influence of pregnancy and hormonal treatment on mammary carcinogenesis. British journal of cancer.

[15] Russo J, Balogh GA, Russo IH (2008). Full-term pregnancy induces a specific genomic signature in the human breast. Cancer epidemiology and prevention Biomarkers.

[16] Asztalos S, Gann PH, Hayes MK, Nonn L, Beam CA, Dai Y, Wiley EL, Tonetti DA (2010). Gene expression patterns in the human breast after pregnancy. Cancer prevention research (Phil).

[17] Ghosh S, Gu F, Wang CM, Lin CL, Liu J, Wang H, Ravdin P, Hu Y, Huang TH, Li R (2014). Genome-wide DNA methylation profiling reveals parity-associated hypermethylation of FOXA1. Breast cancer research and treatment.

[18] Bernardo GM, Keri RA (2012). FOXA1: a transcription factor with parallel functions in development and cancer. Bioscience reports.

[19] Gascard P, Bilenky M, Sigaroudinia M, Zhao J, Li L, Carles A, Delaney A, Tam A, Kamoh B, Cho S, Griffith M, Chu A, Robertson G, Cheung D, Li I, Heravi-Moussavi A, Moksa M, Mingay M, Hussainkhel A, Davis B, Nagarajan RP, Hong C, Echipare L, O'Geen H, Hangauer MJ, Cheng JB, Neel D, Hu D, McManus MT, Moore R, Mungall A, Ma Y, Plettner P, Ziv E, Wang T, Farnham PJ, Jones SJ, Marra MA, Tlsty TD, Costello JF, Hirst M (2015). Epigenetic and transcriptional determinants of the human breast. Nature communications.

[20] Hurtado A, Holmes KA, Ross-Innes CS, Schmidt D, Carroll JS (2011). FOXA1 is a key determinant of estrogen receptor function and endocrine response. Nature genetics.

[21] Carroll JS, Liu XS, Brodsky AS, Li W, Meyer CA, Szary AJ, Eeckhoute J, Shao W, Hestermann EV, Geistlinger TR, Fox EA, Silver PA, Brown M (2005). Chromosome-wide mapping of estrogen receptor binding reveals long-range regulation requiring the forkhead protein FoxA1. Cell.

[22] Watters RJ, Benos PV, Oesterreich S (2012). To bind or not to bind-FoxA1 determines estrogen receptor action in breast cancer progression. Breast cancer research.

[23] Izadi P, Noruzinia M, Fereidooni F, Hosseini ZM, Kamali F (2014). Epigenetic marks in estrogen receptor alpha CpG island correlate with some reproductive risk factors in breast cancer. Molecular biology reports.

[24] Daraei A, Izadi P, Khorasani G, Nafissi N, Naghizadeh MM, Younosi N, Meysamie A, Mansoori Y, Bastami M, Tavakkoly-Bazzaz J (2017). Epigenetic changes of the ESR1 gene in breast tissue of healthy women: A missing link with breast cancer risk factors?. Genetic testing and molecular biomarkers.

[25] Watts P (2001). Extraction of DNA from tissue: high salt method.

[26] Taiwo O, Wilson GA, Morris T, Seisenberger S, Reik W, Pearce D, Beck S, Butcher LM (2012). Methylome analysis using MeDIP-seq with low DNA concentrations. Nature protocols.

[27] Jerry DJ, Makari-Judson G, Crisi GM, Dunphy KA (2013). Pregnancy offers new insights into mechanisms of breast cancer risk and resistance. Breast cancer research.

[28] Nair SS, Coolen MW, Stirzaker C, Song JZ, Statham AL, Strbenac D, Robinson MD, Clark SJ (2011). Comparison of methyl-DNA immunoprecipitation (MeDIP) and methyl-CpG binding domain (MBD) protein capture for genome-wide DNA methylation analysis reveal CpG sequence coverage bias. Epigenetics.

[29] Huh SJ, Clement K, Jee D, Merlini A, Choudhury S, Maruyama R, Yoo R, Chytil A, Boyle P, Ran FA, Moses HL, Barcellos-Hoff MH, Jackson-Grusby L, Meissner A, Polyak K (2015). Age-and pregnancy-associated DNA methylation changes in mammary epithelial cells. Stem cell reports.

[30] Pal B, Bouras T, Shi W, Vaillant F, Sheridan JM, Fu N, Breslin K, Jiang K, Ritchie ME, Young M, Lindeman GJ, Smyth GK, Visvader JE (2013). Global changes in the mammary epigenome are induced by hormonal cues and coordinated by Ezh2. Cell reports.

[31] Colditz GA, Rosner BA, Chen WY, Holmes MD, Hankinson SE (2004). Risk factors for breast cancer according to estrogen and progesterone receptor status. Journal of the national cancer institute.

[32] Santucci-Pereira J, George C, Armiss D, Russo IH, Vanegas JE, Sheriff F, de Cicco RL, Su Y, Russo PA, Bidinotto LT, Russo J (2013). Mimicking pregnancy as a strategy for breast cancer prevention. Breast cancer management.

